# An evaluation of evidence-based paediatric injury prevention policies across Canada

**DOI:** 10.1186/s12889-015-1986-9

**Published:** 2015-07-25

**Authors:** Alison K. Macpherson, Mariana Brussoni, Pamela Fuselli, Tara Middaugh-Bonney, Shannon Piedt, Ian Pike

**Affiliations:** 337 Bethune College, York University, 4700 Keele St, Toronto, ON M3J 1P3 Canada; Department of Pediatrics, University of British Columbia, F508-4480 Oak Street, Vancouver, BC V6H 3 V4 Canada; Parachute Canada, 150 Eglinton Avenue East, Suite 300, Toronto, ON M4P 1E8 Canada; Georgian College, One Georgian Drive, Barrie, ON L4M 3X9 Canada; British Columbia Injury Research and Prevention Unit, F508-4480 Oak Street, Vancouver, BC V6H 3 V4 Canada

**Keywords:** Injury prevention, Policy, Legislation, Child, Adolescent

## Abstract

**Background:**

Policies to reduce injury among Canadians can be controversial and there is variability in the enactment of injury prevention laws across the country. In general, laws are most effective when they are based on good research evidence, supported by widespread public awareness and education, and maintained by consistent enforcement strategies. The purpose of this study was to document and compare key informants’ perceptions of the quality, awareness, and enforcement of three evidence-based paediatric injury prevention policies (bicycle helmet legislation, child booster seat legislation, graduated driver licensing) among Canadian provinces and territories.

**Methods:**

We identified best practices related to each policy, then developed an online survey to ascertain the extent to which each jurisdiction’s policy aligned with best practices, whether experts believed that the public was aware of the policy and whether it was enforced. The survey was distributed using a snowball sampling strategy to key informants across Canada.

**Results:**

Thirty-eight key informants responded to the bicycle helmet survey, with 73 and 35 key informants for the booster seat and graduated driver licensing surveys, respectively. Respondent’s perceptions of the policies varied substantially. Key informants indicated that residents are not always aware of legislation, and legislation is not consistently enforced. These results suggest that child health policy is not always guided by evidence.

**Conclusions:**

There was variation between evidence and the policies related to paediatric injury prevention among Canadian provinces and territories. Experts generally rate their policies more highly when they align with evidence and best practice. There is room for improvement and harmonization of injury prevention policies.

**Electronic supplementary material:**

The online version of this article (doi:10.1186/s12889-015-1986-9) contains supplementary material, which is available to authorized users.

## Background

Experts have long advocated for the three “E’s” of injury prevention, which include education, engineering, and enforcement (of policy). According to Pearn et al., “History has shown that safety legislation – if based on sound research-based evidence, if introduced with widespread community consensus, and if followed by regular compliance inspection, and maintained by penalties for defaulters - is the most effective form of injury prevention” [1]. Rivara et al. concluded that legislation is one of the most effective methods for injury control, leading to many policies having been adopted with a goal to prevent injuries [2]. The term policy is most often defined as ‘government action’ [3], and more specifically, ‘an action which employs governmental authority to commit resources in support of preferred values’ [4]. Injury prevention examples include, seatbelt legislation, drinking and driving laws, and child-resistant covers for medication containers. Many of these policies have been evaluated and found to be effective, such as laws related to bicycle helmets, vehicle booster seat requirements, and graduated driver licensing, which have robust evidence of effectiveness in preventing paediatric injuries [5–9]. Despite scientific evidence, policies are not homogeneous within and between jurisdictions, and the development and enforcement of injury prevention legislation can be controversial. Individuals often do not see themselves as stakeholders for the benefits of injury prevention legislation, and opponents often argue for personal freedom and the relative cost of enforcing laws versus their benefits [1]. Likewise, enacted legislation can be challenged, rescinded or repealed [10].

Despite evidence that bicycle helmet legislation increases helmet use and prevents head injuries [5], there is considerable opposition from some of the cycling public, and ongoing debate about the need for bicycle helmet laws. Furthermore, helmet laws vary considerably across jurisdictions in scope (all ages vs. children only), mandate (everywhere vs. only on public roads), and enforcement. There is evidence to suggest that all ages helmet laws achieve higher levels of helmet use than laws that apply only to children [11–13], and that enforcing laws through fines and tickets results in increased helmet use [14, 15].

Booster seat legislation contains several components including stipulations regarding the age and weight of the occupant, the location of the booster seat in the back seat of the vehicle, legislated exemptions, driver responsibility, non-compliance actions, and incentive programs. While evidence suggests that the appropriate use of child passenger safety seats decreases the risk for injury, the consistent or correct use of these devices in Canada remains low [16]. A study conducted by Snowdon et al. observed 13,500 children (aged birth-9 years old) in 10,084 vehicles across Canada in 2006 [17]. This research showed that 60.5 % of children were restrained in the correct safety seat overall, ranging from 39 % in New Brunswick to 64 % in Alberta. When stratified by age group, the lowest rate of correct use of safety seats was among school-aged children (4–8 years), with 19.6 % correctly restrained in booster seats. The majority of these children (63.1 %) were restrained in adult seat belts. Because of this, researchers advocate for widespread public awareness campaigns, child safety seat technician training programs, child safety seat clinics, and child booster seat legislation [16].

During the early 1970’s, researchers began discussing ways to reduce the risks associated with inexperienced and/or young drivers. “The idea was that [policy makers] could somehow intervene prior to licensure and make crash involvement less likely” [18]. Graduated driver licensing (GDL) was one policy that was introduced that implemented driving restrictions to gradually expose young drivers to the challenges of driving. Some of the elements included are a learner’s permit stage where new drivers must drive with a licensed driver in the car, and a requirement for a zero blood alcohol content, as well as restriction regarding the number of passengers. GDL is now in effect in almost every province, territory and state in North America [9].

The purpose of this study was to document and compare expert stakeholders’ perceptions of the quality, public awareness, and enforcement of three evidence-based paediatric injury prevention policies (bicycle helmet legislation, booster seat legislation, GDL) across Canadian provinces and territories. The specific objectives of this study were to: 1) evaluate the three paediatric injury prevention policies to determine how well they aligned with the best available evidence, and 2) assess how the quality, public awareness and enforcement of the policies were perceived by expert key informants within the jurisdiction in which they were enacted.

## Methods

We followed the methods detailed by Mâsse et al., who ranked physical education policies using a classification system based on the different policies related to physical education within school systems [19], and subsequently school nutrition programs. [20] Based on best practices identified through published studies and reviews, and expert opinion from researchers, practitioners, and policy makers at a national level, survey questions were developed. For example, helmet use has been shown to be higher in areas with legislation that applies to all ages compared to children alone [13], so the age group affected by the law was one of the questions included in the survey related to bicycle helmet legislation. A summary of the best practice elements for each type of legislation and the provinces/territories that include those elements is listed in Table 1.

**Table 1:** Best Practices Summary Table (Provincial/Territorial regulation as of 2009, except where otherwise indicated)

		BC	AB	SK	MB	ON	QC	NS	NB	NL	PEI	YK	NWT	NU
Bicycle helmet	All Ages	✓	X	n/a	X	X	n/a	✓	✓	n/a	✓	n/a	n/a	n/a
	All Roadways	✓	✓	n/a	✓	✓	n/a	✓	✓	n/a	✓	n/a	n/a	n/a
					(2013)									
Booster seat	9 years of age & height 145 cm	✓	n/a	X	✓	✓	X	✓	✓	n/a	✓	n/a	n/a	n/a
					(2013)									
	Weight 18–36 kg	✓	n/a	✓	✓	✓	✓	✓	✓	n/a	✓	n/a	n/a	n/a
				(2014)	(2013)									
	Public education programs	✓	n/a	✓	✓	✓	✓	✓	✓	n/a	✓	n/a	n/a	n/a
	Under 12 seated in back seat	X	n/a	X	X	X	X	X	X	n/a	X	n/a	n/a	n/a
	No vehicle exemptions (e.g. for medical vehicles or public/school buses)	X	n/a	X	X	X	X	X	X	n/a	X	n/a	n/a	n/a
	Driver responsibility	✓	n/a	X	✓	✓	X	✓	✓	n/a	✓	n/a	n/a	n/a
					(2013)									
	Non-compliance penalties	✓	n/a	✓	✓	✓	✓	✓	✓	n/a	✓	n/a	n/a	n/a
				(2014)	(2013)									
	Incentive programs	✓	n/a	✓	X	✓	X	✓	X	n/a	X	✓	n/a	n/a
	(e.g. providing free or discounted booster seats to families who qualify)			(2014)										
GDL	Learner stage	✓	✓	✓	✓	✓	✓	✓	✓	✓	✓	✓	✓	n/a
	Intermediate stage	✓	✓	✓	✓	✓	✓	✓	✓	✓	✓	✓	✓	n/a
	BAC restrictions	✓	✓	✓	✓	✓	✓	✓	✓	✓	✓	✓	✓	n/a
	Nighttime driving curfew	✓	✓	X	X	✓	X	X	X	✓	✓	✓	✓	n/a
	Passenger restrictions	✓	X	X	X	X	X	✓	✓	✓	✓	✓	✓	n/a
	Cell phone restrictions	✓	X	✓	X	X	X	X	X	X	✓	X	X	n/a
	(Some provinces have now enacted cell phone restrictions outside of GDL. Those are not included here.)													
	‘L’ and ‘N’ sign plates	✓	X	X	X	X	X	X	X	✓	✓	✓	X	n/a
	No time discount for driver education	X	✓	✓	✓	X	X	X	X	X	✓	✓	✓	n/a

Legend:

✓ Regulation included within the law of this province/territory

X Regulation not included within the law of this province/territory

n/a Province/Territory does not have a law

The survey included a rating scale to assess policies, and components within the policies in terms of: i) alignment with research evidence and best practices, ii) perceived public awareness of the existence of the policy and its components, and iii) the degree to which the policy was enforced. Acknowledging that different key informant experts would be required to rate each of the policies, separate surveys were created for each of the three policies under study. Complete versions of each survey are included as an additional file (see Additional file 1). Ethical approval for the study was obtained from the University of British Columbia – Children’s and Women’s Health Centre of BC Research Ethics Board, reference number CW08-0231 / H08-00834.

The research team, in consultation with national level practitioners and policy-makers, generated a list of provincial leadership positions related to the three policies, as well as a list of experts in bicycle helmet, child seat safety and GDL injury prevention research or practice. The Standing Committee on Road Safety Research and Policies of the Canadian Council of Motor Transport Administrators was approached and agreed to distribute the appropriate survey link by email to all members of their committee. Using a snowball technique, individuals who held each provincial leadership position within insurance, government (Solicitor General, Deputy Registrar of Motor Vehicles, etc.), law enforcement (Chief of Police, Inspector for RCMP Provincial Traffic Services, etc.) or injury prevention (policy analyst, SMARTRISK, etc.) were invited to be survey respondents. The invitation email included the statement “If you are not the correct person to provide expertise, please forward the survey link below to a professional colleague in your province who understands your provincial [booster seat] law”. All surveys were available in English and French, and were posted using online survey tools. Informed consent for participation in the study was obtained from experts via an online consent form preceding the survey.

The snowball sampling started with the list of 80 experts as noted above; between 5 and 11 experts were identified for each province, one expert each for the Yukon and Northwest Territories and no experts identified for Nunavut. As initial response rates for the bicycle helmet legislation and GDL surveys were lower than anticipated, a more extensive list of 153 experts was identified for the booster seat survey; between 8 and 22 experts for each province, and 2–3 experts for each Territory. Each potential expert received the email request to participate together with an electronic link to the survey, followed by a reminder email one month later. Alignment with best practices and degree of enforcement were rated using a Likert type scale where 0 represented ‘not good’, 5 represented ‘average’ and 10 represented ‘extremely good’. The level of public awareness was rated by the respondent’s estimate of the proportion (percent) of the population within the jurisdiction that was aware of the policy and its components. Demographic questions were the same for each survey.

All respondents were asked to rate the awareness of the specific elements of each policy within the general population in that province or territory, and to rate the effectiveness and enforcement of that policy. Specific questions for bicycle helmet legislation included: i) the age range of cyclists to which helmet legislation applied [11, 12], ii) perceived enforcement of the legislation [14], and iii) where the legislation was applied (everywhere vs. on roadways only) [15].

For booster seat laws, the survey focused on enforcement and varying components of booster seat legislation. Well documented best practices include occupant weight stipulations, stipulations for placement of the booster seat and occupant in the vehicle back seat, whether some vehicles (e.g., taxis) were exempt from the legislation, driver responsibility, non-compliance actions, and incentive programs, all of which were included in the survey questions [21–24]. In addition, the survey asked whether there were appropriate booster seat use guidance clinics and/or telephone support lines for parents and caregivers in the province or territory [25].

For GDL, the survey focused on: issuance of a learner permit, an intermediate licensing stage, restrictions on driver blood alcohol concentration, seatbelt use requirement, night time driving curfew, restrictions regarding the number of passengers, cell phone use restrictions, “L” (learner) and ”N” (new driver) signs/plates, and time to full licensure discount for driver education completion [26–29]. Each component was individually rated in terms of alignment with best practices, public awareness, and enforcement.

## Results and discussion

There were a total of 146 responses to the surveys, with the majority of people completing the booster seat survey (73) followed by bicycle helmet (38) and GDL (35). Completed surveys were received from all Canadian provinces and the Yukon Territory. However, not all provinces and territories had enacted each policy at the time the surveys were conducted, and this is indicated in the results tables below as ‘n/a’. All respondents correctly identified the presence or absence of each of the laws.

Table 2 compares the general evaluation of each of the laws by province or territory. Table 3 examines the perceived public awareness of policies and selected elements within policies, and Table 4 focuses on the perceived enforcement of selected elements of each policy. The alignment of each province or territory’s policies with best practice were based on the Canadian Pediatric Society’s assessment of bicycle helmet and booster seat legislation, and the Traffic Injury Research Foundation’s assessment of GDL [26, 30, 31]. In general, policies that were concordant with best practices were rated higher in terms of public awareness and enforcement than those that were not. GDL generally rated higher than the two other policies, while bicycle helmet laws for children only were rated lower. Enforcement was perceived as much higher for GDL than for the other policies.

**Table 2:** Overall rating of each policy by province or territory and number of respondents (Mean (SD) based on a Likert type scale where 0 = not good, 5 = average, and 10 = extremely good)

	BC	AB	SK	MB	ON	QC	NS	NB	NL	PEI	YK
Bicycle helmet	9.8 (0.5)	3.0 (2.3)	n/a	n/a	7.0 (4.2)	n/a	10.0 (0)	9.3 (1.2)	n/a	7.7 (2.5)	n/a
Bicycle helmet (number of respondents)	n = 3	n = 3			n = 4		n = 4	n = 3		n = 3	
Booster seat	7.7 (3.2)	n/a	n/a	n/a	5.1 (2.3)	6.6 (3.0)	7.0 (1.7)	7.3 (1.7)	5.0 (4.2)	n/a	n/a
Booster seat (number of respondents)	n = 3				n = 17	n = 4	n = 2	n = 8		n = 3	
GDL	9.0 (0)	3.7 (2.1)	7.7 (0.6)	5.3 (3.5)	8.7 (2.3)	8.0 (0)	5.3 (1.5)	8.0 (0)	6.5 (2.1)	8.5 (0.7)	6.5 (1.4)
GDL (number of respondents)	n = 6	n = 3	n = 3	n = 3	n = 3	n = 2	n = 4	n = 3	n = 2	n = 2	n = 2

**Table 3:** Perceived proportion (%) of the public that is aware of each policy by province or territory

	BC	AB	SK	MB	ON	QC	NS	NB	NL	PEI	YK
Bicycle helmet law general awareness	72.5	50.0	n/a	n/a	36.7	n/a	85.0	63.3	n/a	73.3	n/a
Booster seat law general awareness	67.5	n/a	n/a	n/a	60.0	57.5	60.2	62.1	50.0	72.5	n/a
GDL Learner’s Permit	90.0	86.7	70.1	70.0	96.7	55.0	80.0	86.7	95.0	100	65.0

**Table 4:** Perceived enforcement of selected elements of each policy by province or territory (Mean (SD) based on a Likert type scale where 0 = not good, 5 = average, and 10 = extremely good)

	BC	AB	SK	MB	ON	QC	NS	NB	NL	PEI	YK
Bicycle helmet (generally)	3.0 (0)	1.7 (0.6)	n/a	n/a	5.3 (5.5)	n/a	6.5 (1.0)	1.7 (2.1)	n/a	5.3 (2.5)	n/a
Booster seat Age and Weight Stipulations	7.3 (3.1)	n/a	n/a	n/a	6.1 (2.6)	8.0 (2.0)	9.0 (0)	8.3 (1.3)	n/a	8.0 (1.9)	n/a
GDL Learner Permit	9.3 (0.8)	8.5 (1.1)	7.3 (1.7)	6.7 (2.1)	8.3 (2.4)	6.0 (1.0)	7.3 (1.8)	10.0 (0)	8.0 (1)	10.0 (0)	8.0 (0)
GDL Blood Alcohol Restrictions	10.0 (0)	6.0 (3.6)	9.3 (0.6)	9.3 (1.2)	8.3 (2.9)	9.0 (0)	4.3 (5.1)	10.0 (0)	8.55 (0.7)	10.0 (0)	6.0 (4.2)
GDL Cell Phone Restrictions	4.0 (5.7)	2.0 (0)	n/a	n/a	n/a	9.0 (0)	7.8 (4.5)	1.0 (1.4)	6.5 (2.1)	8.0 (0)	n/a

In addition to the numeric ranking, the surveys provided respondents with the opportunity to add further comments. Respondents wrote comments related to enforcement in all three surveys. Examples included: “the lack of exemptions is good in the legislation, but the lack of enforcement and education make the inclusions moot” (booster seat survey), “police enforcement is low at the moment…but definitely in need of attention” (booster seat survey), “L and N sign plates are not a high priority for police enforcement although it is the law” (GDL survey) and “we need more energy and time to law enforcement” (bicycle helmet survey). In addition to the results in Table 3 regarding public awareness of the law, one respondent from the bicycle helmet legislation survey commented, “no surveys have been completed to determine the percentage of the public who are aware of the helmet law. Anecdotal observations from our marketing planners range from 30 to 70 % depending on the region…”. With respect to the existence of booster seat guidance clinics or telephone hotlines, a respondent wrote “public health units and other not for profit organizations try to support this issue, but the demand is so great that the need is never met”. Another wrote, “Alberta needs booster seat legislation. There also needs to be a means of getting training for parents to properly use child restraint assemblies. The current programs are not widely publicized and the free clinics have been cancelled”. Finally, when asked to comment on booster seat legislation overall, one Albertan response was “no legislation to rate, a deplorable situation”.

There is substantial variation within and between provinces related to injury prevention policies, and the perception among key informant experts of their alignment with research evidence and best practices, public awareness and enforcement. The policy that is most broadly adopted and enforced is GDL, and it also received the highest ratings on the policy’s alignment with evidence and best practice, and public awareness. Both bicycle helmet and booster seat legislation were less consistent across the jurisdictions. Some respondents were clearly concerned that the policies had not been enacted in their jurisdiction, while others were disconcerted by the lack of enforcement. There appears to be an association between the concordance with best practice and the ranking of the policy by those in each jurisdiction. These results mirror the Canadian Paediatric Society’s “Are we doing enough?” report, which calls for the implementation and improvement of booster seat legislation and all ages bicycle helmet legislation in all provinces and territories, so that policies better align with evidence and best practice [31]. Interestingly, none of the respondents cited public opposition, nor costs associated with enforcement of any of the three policies as concerns in their jurisdiction.

The higher ratings for legislation that aligned with best practice may be due, at least in part, to the fact that the survey was based on research evidence and best practice. However, legislation that aligned with evidence and best practice also received higher ratings of public awareness. For example, the bicycle helmet law in Nova Scotia met most of the requirements for best practice, and had the highest perceived public awareness. It is possible that when the law applies to adults as well as children that everyone is perceived as being more aware of the law. Further, GDL had the highest ratings for awareness, but that may be due to requirements for testing and examination related to obtaining learner’s permits and driver licences. Although the police enforce the GDL laws, the licensing practices are regulated by the Ministry of Transport.

These results reinforce previous findings that child health policy is not always guided by evidence [32]. The results in Tables 3 and 4 along with respondent comments illustrate that residents are not always aware of legislation, and legislation is not consistently enforced. Previous research has established that injury prevention legislation that is based on good research evidence is effective, however, it is most effective when supported by widespread public awareness and education, and maintained by consistent enforcement strategies [1].

A recent example of using the combination of policy, awareness and enforcement of injury prevention legislation occurred in the province of British Columbia with the introduction in 2010 of its Immediate Roadside Prohibition (IRP) legislation that aimed to deter drinking drivers by increasing the certainty, severity and swiftness of sanctions. The legislation was passed to help police and the courts more effectively process drinking drivers and increase the likelihood of apprehension and punishment. The IRP legislation supplemented laws under the Criminal Code of Canada, which had more severe penalties but also lower probability of punishment. Together with extensive public awareness and education campaigns informing the public of the new law and the enhanced enforcement strategy, the IRP resulted in significant reductions in mortality, serious injuries and property damage. In an evaluation of the impact of the IRP legislation, which accounted for the 15-year pre-law downtrend in alcohol related motor vehicle crash fatalities and injuries, Macdonald, et al. reported a 40 % reduction in fatalities from alcohol-related crashes, a 23 % decline in injuries and a reduction in property damage of 19.5 % [33]. Key to the success of the IRP legislation was the multi-sector support and the combination of policy and enforcement. Based upon this successful approach, researchers, advocates, and policy-makers are urged to continue to collaborate to improve child injury prevention policies across the country and to employ a multi-sectoral approach to development, implementation and enforcement.

### Strengths and limitations

This is the first study to compare and evaluate evidence-based injury prevention policies across jurisdictions by identifying experts in each province to evaluate policies. However, their opinions may not reflect those of the general population. The key informant survey technique employed is subjective and based upon participant expertise and experience. It is possible that the results would be different had a different group of key informants participated. Because of the anonymous nature of the survey, we do not know whether it was the invited key informant, or a subsequent individual invited by them, that actually responded to the survey. However, as we communicated the importance that the survey be completed by those with expertise around the policy in their jurisdiction and knowledgeable in the field, we assume that any subsequent invitees had been identified as having those qualities.

Further, the responses are based on key informant expertise and perceptions, and may not reflect actual awareness and enforcement of these laws. However, policy analysis is often based on the opinions of policy-makers and those responsible for the implementation and enforcement of policies.

Finally, this study was conducted in Canada and the results may not be generalizable to other jurisdictions.

## Conclusion

There is variation between best available evidence and the policies related to paediatric injury prevention among Canadian provinces and territories. In general, experts rate their policies more highly when they align with research evidence and best practice. There is room for substantial improvement and harmonization of injury prevention policies across Canada.

## Additional file

Additional file 1:
**A document of the surveys that were sent to experts as part of the study methods.** This file includes surveys in French and English for: i) bicycle helmet legislation, ii) graduated driver licensing regulations, and iii) booster seat legislation.

## Abbreviations

GDL: Graduated driver licensing
RCMP: Royal Canadian Mounted Police
IRP: Immediate roadside prohibition
L: Learner
N: New driver
CIHR: Canadian Institutes of Health Research
BC: British Columbia
AB: Alberta
SK: Saskatchewan
MB: Manitoba
ON: Ontario
QC: Quebec
NS: Nova Scotia
NB: New Brunswick
NL: Newfoundland and Labrador
PEI: Prince Edward Island
YK: Yukon
NWT: Northwest Territories
NU: Nunavut
SD: Standard deviation
