# Supplementary material for: An evaluation of evidence-based paediatric injury prevention policies across Canada
Source: BMC Public Health. 2015 Jul 25;15:707. doi: 10.1186/s12889-015-1986-9 (PMC4514983; doi:10.1186/s12889-015-1986-9)
Supplement: Additional file 1: — A document of the surveys that were sent to experts as part of the study methods. This file includes surveys in French and English for: i) bicycle helmet legislation, ii) graduated driver licensing regulations, and iii) booster seat legislation. [file 12889_2015_1986_MOESM1_ESM.pdf]

Bicycle Helmet Law

1. Introduction/Demographics

The Canadian Injury Indicators Team, funded by the Canadian Institutes of Health Research (CIHR), is attempting to understand the best practice elements that are outlined in bike helmet laws in different provinces in Canada. The objective of this survey is to evaluate the components of provincial bicycle helmet legislation, including enforcement and public awareness, against evidence-based best practices in bicycle helmet legislation. The results of this survey will be used to improve the effectiveness of provincial bicycle helmet legislation in reducing bicycle-related injuries and fatalities in Canada.

1. As it relates to road safety, do you regard yourself as a practitioner, policy maker, researcher or physician?

- [ ] Practitioner
- [ ] Policy Maker
- [ ] Researcher
- [ ] Physician

Other (please specify)

____

2. In which province do you work?

- [ ] British Columbia
- [ ] Alberta
- [ ] Saskatchewan
- [ ] Manitoba
- [ ] Ontario
- [ ] Quebec
- [ ] New Brunswick
- [ ] Nova Scotia
- [ ] Prince Edward Island
- [ ] Newfoundland/Labrador

Bicycle Helmet Law

3. Please select the choice that best corresponds to you

- [ ] Have a special interest in the area of bicycle helmet laws
- [ ] Primarily work in the area of road safety
- [ ] Have a job related to the area of road safety and also have a special interest in bicycle helmet laws

Other (please specify)

4. What proportion of your work day do you spend on issues related to bicycle helmet laws?
Note: 0 equals NO time spent on this issue, 5 equals half of work time spent on this issue, 10 equals all work time spent on this issue

	0	1	2	3	4	5	6	7	8	9	10
Time spent on this issue	○	○	○	○	○	○	○	○	○	○	○

5. Which sector do you work in?

- [ ] Government
- [ ] University/college
- [ ] Hospital and/or public health
- [ ] Recreation and sport

Other (please specify)

6. What is the scope of your work?

- [ ] Provincial
- [ ] Federal
- [ ] Both

Other (please specify)

7. What is your job title?

2. Rating your provincial bicycle helmet law

Bicycle Helmet Law

1. Does your province have a bicycle helmet law?

- ◯ Yes
- ◯ No
- ◯ Don't know

2. Is there public awareness/education that this law exists?

- ◯ Yes
- ◯ No
- ◯ Don't know
- ◯ N/A

3. If yes, in your best estimate, what percentage of the public is currently aware of the law?

	0%	10%	20%	30%	40%	50%	60%	70%	80%	90%	100%	N/A
%	◯	◯	◯	◯	◯	◯	◯	◯	◯	◯	◯	◯

3. Who does your bicycle helmet law apply to?

1. Does your province's bicycle helmet law apply to everyone (all ages)?

- ◯ Yes
- ◯ No
- ◯ Don't know

2. If no, does your province’s law have an age of 18 and under it applies to?

- ◯ Yes
- ◯ No
- ◯ Don't know
- ◯ N/A

3. How would you rate your province's bicycle helmet law with regards to the age that the law applies to?

Best practice requires the bicycle helmet law to apply to all ages.

The overall rating scale is 0 = not good, 5 = average, and 10 = extremely good.

	0	1	2	3	4	5	6	7	8	9	10
Age law applies to	◯	◯	◯	◯	◯	◯	◯	◯	◯	◯	◯

Further comments (optional)

4. Enforcement of bicycle helmet law

1. In your experience or expert opinion, your province’s bicycle helmet law is:

- ◯ Strictly enforced
- ◯ Strongly enforced
- ◯ Minimally enforced
- ◯ Don't know
- ◯ N/A

2. How would you rate your province’s enforcement of its bicycle helmet law? The overall rating scale is 0 = not good, 5 = average, and 10 = extremely good.

	0	1	2	3	4	5	6	7	8	9	10	N/A
Enforcement	◯	◯	◯	◯	◯	◯	◯	◯	◯	◯	◯	◯

Further comments (optional)

5. Types of roads that bicycle helmet law applies to

Bicycle Helmet Law

1. Does your province's bicycle helmet law apply to all road ways and all types of roads?

- ◯ Yes
- ◯ No
- ◯ Don't know
- ◯ N/A

2. If no, does your province's law apply only to public roads?

- ◯ Yes
- ◯ No
- ◯ Don't know
- ◯ N/A

3. How would you rate your province's bicycle helmet law with regards to the road ways the law applies to?

Best practice requires the bicycle helmet law to apply to all roadways and all types of roads.

The overall rating scale is 0 = not good, 5 = average, and 10 = extremely good.

	0	1	2	3	4	5	6	7	8	9	10	N/A
Types of roads	◯	◯	◯	◯	◯	◯	◯	◯	◯	◯	◯	◯

Further comments (optional)

6. Overall

1. Considering your responses on this survey, please rate your province's bicycle helmet law. The overall rating scale is 0 = not good, 5 = average, and 10 = extremely good.

	0	1	2	3	4	5	6	7	8	9	10
Overall	◯	◯	◯	◯	◯	◯	◯	◯	◯	◯	◯

Further comments (optional)

Bicycle Helmet Law

7. Thank you

Thank you for contributing to an understanding of bicycle helmet laws in Canada. Your participation in the survey is greatly appreciated!

La loi sur le port du casque de vélo

1. Introduction/démographie

L'Équipe Canadienne sur les indicateurs des blessures (Canadian Injury Indicators Team), financée par les Instituts de Recherche en Santé du Canada(IRSC), tente de mieux comprendre quelles sont les composantes des meilleures pratiques associées aux lois sur le port du casque de vélo dans les différentes provinces du Canada. L'objectif de cette enquête est d'évaluer les composants de la législation provinciale sur le port du casque de vélo, incluant son application et la sensibilisation du public à l'endroit des pratiques exemplaires concernant la législation sur le port du casque de vélo. Les résultats de cette enquête pourront contribuer à améliorer l'efficacité de la législation provinciale sur le port du casque de vélo en réduisant les blessures et les décès associés à la pratique de la bicyclette au Canada.

1. En ce qui concerne les questions de sécurité routière, vous considérez-vous comme un praticien, un décideur politique, un chercheur ou un médecin?

- [ ] Praticien
- [ ] Décideur politique
- [ ] Chercheur
- [ ] Médecin

Autre (spécifiez svp)

____

2. Dans quelle province travaillez-vous?

- [ ] Colombie-Britannique
- [ ] Alberta
- [ ] Saskatchewan
- [ ] Manitoba
- [ ] Ontario
- [ ] Québec
- [ ] Nouveau-Brunswick
- [ ] Nouvelle-Écosse
- [ ] Île-du-Prince-Édouard
- [ ] Terre-Neuve et Labrador

La loi sur le port du casque de vélo

3. Cochez la phrase qui vous décrit le mieux:

- [ ] Êtes intéressé particulièrement au domaine des lois sur le port du casque de vélo
- [ ] Travaillez principalement dans le secteur de la sécurité routière
- [ ] Avez un emploi en lien avec le domaine de la sécurité routière et avez également un intérêt particulier pour des lois sur le port du casque de vélo

Autre (spécifiez svp)

4. Quelle proportion de vos journées de travail consacrez-vous sur des questions relatives aux lois sur le port du casque de vélo?
Note : 0 égale AUCUNE heure passée sur cette question, 5 égale moitié de temps de travail dépensée sur cette question, 10 égale toutes les heures de travail dépensée sur cette question

	0	1	2	3	4	5	6	7	8	9	10
Temps consacré à cette question	○	○	○	○	○	○	○	○	○	○	○

5. Dans quel secteur travaillez-vous?

- [ ] Gouvernement
- [ ] Universitaire
- [ ] Hôpital et/ou santé publique
- [ ] Loisir et sport

Autre (spécifiez svp)

6. À quel niveau territorial travaillez-vous?

- [ ] Provincial/territorial
- [ ] Fédéral
- [ ] Tous les deux

Autre (spécifiez svp)

7. Quel est votre titre d’emploi?

2. Évaluation de votre loi provinciale en matière de port du casque de vélo

La loi sur le port du casque de vélo

1. Votre province a-t-elle une loi sur le port du casque de vélo?

- ◯ Oui
- ◯ Non
- ◯ Ne savez pas

2. Y a-t-il une sensibilisation du public / éducation sur le fait que cette loi existe?

- ◯ Oui
- ◯ Non
- ◯ Ne savez pas
- ◯ Ne s'applique pas

3. Si oui, quelle est votre meilleure estimation du pourcentage de la population qui est au courant de la loi ?

	0%	10%	20%	30%	40%	50%	60%	70%	80%	90%	100%	N/A
%	◯	◯	◯	◯	◯	◯	◯	◯	◯	◯	◯	◯

3. À qui votre loi sur le port du casque de vélo s'adresse-t-elle ?

1. Est-ce que la loi sur le port du casque de vélo de votre province s'applique à tous les groupes d'âge?

- ◯ Oui
- ◯ Non
- ◯ Ne savez pas

2. Si non, la loi de votre province s'applique-t-elle aux enfants âgés de moins de 18 ans?

- ◯ Oui
- ◯ Non
- ◯ Ne savez pas
- ◯ Ne s'applique pas

La loi sur le port du casque de vélo

3. Comment évalueriez-vous votre loi provinciale en ce qui concerne les groupes d’âges qui sont ciblés?
(Les pratiques exemplaires suggèrent que la loi sur le port du casque de vélo doit s’appliquer à tous les âges.)

L'échelle d'évaluation globale est 0 = pas très bon, 5 = moyen, et 10 = extrêmement bon.

	0	1	2	3	4	5	6	7	8	9	10
Les groupes d’âges visés par la loi	◯	◯	◯	◯	◯	◯	◯	◯	◯	◯	◯

D'autres commentaires (facultatif)

4. L'application des lois sur le port du casque de vélo

1. Selon votre expérience ou à votre avis, la loi sur le port du casque de vélo de votre province est:

- ◯ Strictement renforcée
- ◯ Fortement renforcée
- ◯ Renforcée d'une façon minimale
- ◯ Ne sait pas
- ◯ Ne s’applique pas

2. Comment évalueriez-vous votre loi provinciale en ce qui concerne son renforcement?

L'échelle d'évaluation globale est 0 = pas très bon, 5 = moyen, et 10 = extrêmement bon.

	0	1	2	3	4	5	6	7	8	9	10	N/A
Renforcement	◯	◯	◯	◯	◯	◯	◯	◯	◯	◯	◯	◯

D'autres commentaires (facultatif)

5. Types de routes aux lesquelles la loi sur le port du casque de vélo s’appli...

La loi sur le port du casque de vélo

1. La loi sur le port du casque de vélo de votre province s'applique-t-elle à toutes les voies et à tous les types de routes?

- ◯ Oui
- ◯ Non
- ◯ Ne sait pas
- ◯ Ne s'applique pas

2. Si non, la loi de votre province s'applique-t-elle seulement aux voies publiques?

- ◯ Oui
- ◯ Non
- ◯ Ne sait pas
- ◯ Ne s'applique pas

3. Comment évalueriez-vous votre loi provinciale en ce qui concerne les types de route auxquelles elle s'applique?

(Les pratiques exemplaires suggèrent que la loi sur le port du casque de vélo doive s'appliquer à tous les types de voies et à tous les types de routes.)

L'échelle d'évaluation globale est 0 = pas très bon, 5 = moyen, et 10 = extrêmement bon.

	0	1	2	3	4	5	6	7	8	9	10	N/A
Toutes les routes	◯	◯	◯	◯	◯	◯	◯	◯	◯	◯	◯	◯

D'autres commentaires (facultatif)

6. Globalement

La loi sur le port du casque de vélo

1. En se basant sur vos réponses ci-dessus, évaluez svp la loi sur le port du casque de vélo de votre province.

L'échelle d'évaluation globale est 0 = pas très bon, 5 = moyen, et 10 = extrêmement bon.

	0	1	2	3	4	5	6	7	8	9	10
Globalement	○	○	○	○	○	○	○	○	○	○	○

D'autres commentaires (facultatif)

7. Merci

Merci d'avoir contribuer à une meilleure compréhension des lois sur le port du casque de vélo au Canada. Votre participation est très appréciée!

Graduated Driver Licensing Regulations

1. Introduction/Demographics

Provinces in Canada have implemented graduated driver licensing (GDL) laws since 1994. However, many of these laws have significant gaps and exemptions in coverage that diminish the protection that all youth require in motor vehicles. Political will and the translation of that will into political and legislative action is essential to ensure the optimization of youth/child, driver/occupant safety. Recognizing this, the Canadian Injury Indicators Team, funded by the Canadian Institutes of Health Research (CIHR), is attempting to understand the best practice elements that are outlined in the GDL laws in different provinces in Canada. The objective of this survey is to evaluate legislative elements against evidence-based best practices in GDL legislation, legislative enforcement and education/awareness efforts. The team will use the survey results to suggest ways of improving the effectiveness of provincial GDL legislation to reduce youth crash-related injuries and fatalities in Canada.

1. As it relates to road safety, do you regard yourself as a practitioner, policy maker, researcher or physician?

- [ ] Practitioner
- [ ] Policy Maker
- [ ] Researcher
- [ ] Physician

Other (please specify)

2. In which province do you work?

- [ ] British Columbia
- [ ] Alberta
- [ ] Saskatchewan
- [ ] Manitoba
- [ ] Ontario
- [ ] Quebec
- [ ] New Brunswick
- [ ] Nova Scotia
- [ ] Prince Edward Island
- [ ] Newfoundland/Labrador

Graduated Driver Licensing Regulations

3. Please select the choice that best corresponds to you

- [ ] Have a special interest in the area of graduated driver licensing (GDL)
- [ ] Primarily work in the area of road safety
- [ ] Have a job related to the area of road safety and also have a special interest in GDL

Other (please specify)

4. What proportion of your work day do you spend on graduated driver licensing?
Note: 0 equals NO time spent on this issue, 5 equals half of work time spent on this issue, 10 equals all work time spent on this issue

	0	1	2	3	4	5	6	7	8	9	10
Time spent on this issue	○	○	○	○	○	○	○	○	○	○	○

5. Which sector do you work in?

- [ ] Government
- [ ] University/college
- [ ] Hospital and/or public health
- [ ] Recreation and sport

Other (please specify)

6. What is the scope of your work?

- [ ] Provincial
- [ ] Federal
- [ ] Both

Other (please specify)

7. What is your job title?

2. Learner permit stage

Graduated Driver Licensing Regulations

1. Does your province have a learner permit stage?

- Yes
- No
- Don't know

2. If your answer is yes, then in your experience or expert opinion is the learner permit stage strictly enforced?

- Yes
- No
- Don't know
- N/A

3. In your best estimate, what percentage of the public is currently aware of the learner permit stage?

	0%	10%	20%	30%	40%	50%	60%	70%	80%	90%	100%	N/A
%												

4. Considering enforcement, public awareness, and best practice, how would you rate your province's learner permit stage?

Current best practices for the learner permit stage include:
• a period of at least 12 months
• written permission from a parent or legal guardian
• strict parental supervision at all times
• progressing to the intermediate stage only if citation-free during this stage

The overall rating scale is 0 = not good, 5 = average and 10 = extremely good

	0	1	2	3	4	5	6	7	8	9	10	N/A
Learner permit stage												

Further comments (optional)

3. Intermediate Stage

Graduated Driver Licensing Regulations

1. Does your province have an intermediate stage as part of its graduated driver licensing regulation?

- ◯ Yes
- ◯ No
- ◯ Don't Know

2. If your answer is yes, then in your experience or expert opinion is the intermediate stage strictly enforced?

- ◯ Yes
- ◯ No
- ◯ Don't know
- ◯ N/A

3. In your best estimate, what percentage of the public is currently aware of the intermediate stage?

	0%	10%	20%	30%	40%	50%	60%	70%	80%	90%	100%	N/A
%	◯	◯	◯	◯	◯	◯	◯	◯	◯	◯	◯	◯

4. Considering enforcement, public awareness, and best practice, how would you rate your province's intermediate stage?

Current best practices for the intermediate stage include:
• progressing to the full license stage only if citation-free during learner and intermediate stages
• in the event of an at-fault crash or a conviction, the young driver is required to take a driver education refresher course or a similar driver improvement action before progressing to full license stage
• at least 50 hours (including night time driving) of certified practice through a log book system before completing this stage

The overall rating scale is 0 = not good, 5 = average and 10 = extremely good

	0	1	2	3	4	5	6	7	8	9	10	N/A
Intermediate stage	◯	◯	◯	◯	◯	◯	◯	◯	◯	◯	◯	◯

Further comments (optional)

Graduated Driver Licensing Regulations

4. Blood alcohol concentration

1. Does your province’s GDL law have restrictions for blood alcohol concentration?

- ◯ Yes
- ◯ No
- ◯ Don't know

2. If your answer is yes, then in your experience or expert opinion, are blood alcohol restrictions enforced?

- ◯ Yes
- ◯ No
- ◯ Don't know
- ◯ N/A

3. In your best estimate, what percentage of the public is currently aware of the blood alcohol restrictions?

	0%	10%	20%	30%	40%	50%	60%	70%	80%	90%	100%	N/A
%	◯	◯	◯	◯	◯	◯	◯	◯	◯	◯	◯	◯

4. Considering enforcement, public awareness, and best practice, how would you rate your provincial GDL regulations with regards to blood alcohol restrictions?

Zero tolerance (no alcohol at all while driving) is best practice.

The overall rating scale is 0 = not good, 5 = average and 10 = extremely good

	0	1	2	3	4	5	6	7	8	9	10	N/A
Blood alcohol restriction	◯	◯	◯	◯	◯	◯	◯	◯	◯	◯	◯	◯

Further comments (optional)

5. Seat belt requirement

Graduated Driver Licensing Regulations

1. Does your province’s GDL law have a compulsory seatbelt requirement?

- Yes
- No
- Don't know

2. If your answer is yes, then in your experience or expert opinion is the compulsory seatbelt requirement for young drivers enforced?

- Yes
- No
- Don't know
- N/A

3. In your best estimate, what percentage of the public is currently aware of the compulsory seatbelt requirement?

	0%	10%	20%	30%	40%	50%	60%	70%	80%	90%	100%	N/A
%												

4. Considering enforcement, public awareness, and best practice, how would you rate your provincial GDL regulations with regards to seatbelt requirements?

Best practice requires compulsory seat belt wearing while driving.

The overall rating scale is 0 = not good, 5 = average and 10 = extremely good

	0	1	2	3	4	5	6	7	8	9	10
Seatbelt requirement											

Further comments (optional)

6. Nighttime driving curfew

Graduated Driver Licensing Regulations

1. Does your province’s GDL law have a nighttime driving curfew?

- ◯ Yes
- ◯ No
- ◯ Don't know

2. If your answer is yes, then in your experience or expert opinion is the nighttime driving curfew strictly enforced?

- ◯ Yes
- ◯ No
- ◯ Don't know
- ◯ N/A

3. In your best estimate, what percentage of the public is currently aware of the nighttime driving curfew?

	0%	10%	20%	30%	40%	50%	60%	70%	80%	90%	100%	N/A
%	◯	◯	◯	◯	◯	◯	◯	◯	◯	◯	◯	◯

4. Considering enforcement, public awareness, and best practice, how would you rate your province's GDL regulations with regards to nighttime driving curfew?

Best practice is to have unsupervised driving prohibited from at least 10:00 pm to 5:00 am.

The overall rating scale is 0 = not good, 5 = average and 10 = extremely good

	0	1	2	3	4	5	6	7	8	9	10	N/A
Nighttime driving curfew	◯	◯	◯	◯	◯	◯	◯	◯	◯	◯	◯	◯

Further comments (optional)

7. Passenger restrictions

Graduated Driver Licensing Regulations

1. Does your province's GDL law have passenger restrictions for young drivers?

- ◯ Yes
- ◯ No
- ◯ Don't know

2. If your answer is yes, then in your experience or expert opinion, are passenger restrictions strictly enforced?

- ◯ Yes
- ◯ No
- ◯ Don't know
- ◯ N/A

3. In your best estimate, what percentage of the public is currently aware of the passenger restrictions?

	0%	10%	20%	30%	40%	50%	60%	70%	80%	90%	100%	N/A
%	◯	◯	◯	◯	◯	◯	◯	◯	◯	◯	◯	◯

4. Considering enforcement, public awareness, and best practice, how would you rate your province's GDL regulations with regards to passenger restrictions?

Best practice limits the number of teenage passengers that may accompany the teen driver.

The overall rating scale is 0 = not good, 5 = average and 10 = extremely good

	0	1	2	3	4	5	6	7	8	9	10	N/A
Passenger restrictions	◯	◯	◯	◯	◯	◯	◯	◯	◯	◯	◯	◯

Further comments (optional)

8. Cell phone restrictions

Graduated Driver Licensing Regulations

1. Does your province’s GDL law have cell phone restrictions?

- ◯ Yes
- ◯ No
- ◯ Don't know

2. If your answer is yes, then in your experience or expert opinion, are cell phone restrictions strictly enforced?

- ◯ Yes
- ◯ No
- ◯ Don't know
- ◯ N/A

3. In your best estimate, what percentage of the public is currently aware of the cell phone restrictions?

	0%	10%	20%	30%	40%	50%	60%	70%	80%	90%	100%	N/A
%	◯	◯	◯	◯	◯	◯	◯	◯	◯	◯	◯	◯

4. Considering enforcement, public awareness, and best practice, how would you rate your provincial GDL regulations with regards to cell phone restrictions?

Banning cell phone use and text messaging while driving is best practice.

The overall rating scale is 0 = not good, 5 = average and 10 = extremely good

	0	1	2	3	4	5	6	7	8	9	10	N/A
Cell phone and text messaging ban	◯	◯	◯	◯	◯	◯	◯	◯	◯	◯	◯	◯

Further comments (optional)

9. “L” “N” sign / plates

Graduated Driver Licensing Regulations

1. Does your province's GDL law require use of 'L' and 'N' signs/plates to indicate 'learner' and 'new' drivers?

- Yes
- No
- Don't know

2. If your answer is yes, then in your experience or expert opinion is use of 'L' and 'N' strictly enforced?

- Yes
- No
- Don't know
- N/A

3. In your best estimate, what percentage of the public is currently aware of the 'L' and 'N' requirement?

	0%	10%	20%	30%	40%	50%	60%	70%	80%	90%	100%	N/A
%	○	○	○	○	○	○	○	○	○	○	○	○

4. Considering enforcement and public awareness, how would you rate your provincial GDL regulations with regards to 'L' and 'N' sign/plates?

Best practice is not yet known.

The overall rating scale is 0 = not good, 5 = average and 10 = extremely good

	0	1	2	3	4	5	6	7	8	9	10	N/A
"L" or "N" sign	○	○	○	○	○	○	○	○	○	○	○	○

Further comments (optional)

10. Time discount for driver education

Graduated Driver Licensing Regulations

1. Does your province's GDL regulation shorten the time of the learner or intermediate stage for young drivers who complete a driver education course?

- Yes
- No
- Don't know

2. Considering best practice, how would you rate your provincial GDL regulations with regards to time discount for driver education?

Best practice is to have NO time discount for driver education.

The overall rating scale is 0 = not good, 5 = average and 10 = extremely good

	0	1	2	3	4	5	6	7	8	9	10	N/A
Driver-Ed time discount												

Further comments (optional)

11. OVERALL RATING

1. Considering your responses to this survey, please rate your province's graduated driver licensing regulations overall.

The overall rating scale is 0 = not good, 5 = average and 10 = extremely good

	0	1	2	3	4	5	6	7	8	9	10
Overall rating											

Further comments (optional)

12. Thank you

Thank you for contributing to an understanding of GDL laws in Canada. Your participation in the survey is greatly appreciated!

Programmes d’obtention du permis de conduire par étapes (PCÉ)

1. Introduction/démographie

Les provinces du Canada ont mis en application des lois concernant l’obtention du permis de conduire par étapes (PCÉ) depuis 1994. Cependant, plusieurs de ces lois ont des lacunes et des exemptions significatives qui peuvent diminuer la protection nécessaire dans les véhicules moteurs pour les jeunes . La volonté politique et la transposition de celle volonté dans l'action politique et législative est essentielle pour assurer l'optimisation de la protection des jeunes et la sécurité des conducteurs et des occupants des véhicules. Après avoir fait ce constat, l'équipe canadienne des indicateurs des traumatismes et blessures, financée par les Instituts de recherche en santé du Canada (IRSC), cherche à comprendre les éléments considérés comme des pratiques exemplaires décrits dans les lois de PCÉ dans les différentes provinces canadiennes. L'objectif de cette enquête est d'évaluer les éléments législatifs des PCÉ au regard des pratiques exemplaires basées sur les évidences scientifiques, l’application de la loi ainsi que les efforts de sensibilisation/éducation du public. L'équipe emploiera les résultats de cette enquête pour suggérer des manières d'améliorer l'efficacité de la législation provinciale des PCÉ pour réduire davantage les blessures et la mortalité reliées aux accidents chez les jeunes au Canada.

1. En ce qui concerne la sécurité routière, vous considérez-vous comme un praticien, un décideur politique, un chercheur ou un médecin ?

- ☐ Praticien
- ☐ Décideur politique
- ☐ Chercheur
- ☐ Médecin

Autre (spécifiez svp)

____

2. Dans quelle province travaillez-vous?

- ☐ Colombie-Britannique
- ☐ Alberta
- ☐ Saskatchewan
- ☐ Manitoba
- ☐ Ontario
- ☐ Québec
- ☐ Nouveau-Brunswick
- ☐ Nouvelle-Écosse
- ☐ Île-du-Prince-Édouard
- ☐ Terre-Neuve et Labrador

Programmes d’obtention du permis de conduire par étapes (PCÉ)

3. Sélectionnez s’il vous plaît la case qui vous décrit le mieux:

- ☐ Vous avez un intérêt spécial pour le secteur des lois sur les PCÉ
- ☐ Vous travaillez principalement dans le secteur de la sécurité routière
- ☐ Vous avez un emploi dans le secteur de la sécurité routière et avez également un intérêt spécial pour des lois sur les PCÉ

Autre (spécifiez svp)

4. Quelle proportion de votre journée de travail est consacrée aux PCÉ? Note : 0 égale AUCUNE, 5 égale moitié du temps de travail, 10 égale toutes les heures de travail consacrées au PCÉ.

	0	1	2	3	4	5	6	7	8	9	10
Temps consacré	○	○	○	○	○	○	○	○	○	○	○

5. Dans quel secteur travaillez-vous?

- ☐ Gouvernement
- ☐ Universitaire
- ☐ Hôpital et/ou santé publique
- ☐ Loisir et sport

Autre (spécifiez svp)

6. Quelle est la portée de votre travail?

- ☐ Provinciale/territoriale
- ☐ Fédérale
- ☐ Les deux

Autre (spécifiez svp)

7. Quel est votre titre d’emploi?

2. Le permis d’apprenti conducteur

Programmes d'obtention du permis de conduire par étapes (PCÉ)

1. La loi du PCÉ de votre province, a-t-elle une étape de d'apprenti conducteur?

- ◯ Oui
- ◯ Non
- ◯ Ne sais pas

2. Si oui, sur la base de votre expérience ou de votre expertise, est-ce que l'étape d'apprenti conducteur est strictement imposée?

- ◯ Oui
- ◯ Non
- ◯ Ne sais pas
- ◯ Ne s'applique pas

3. Selon votre meilleure estimation, quel pourcentage du public connaît l'étape d'apprenti conducteur?

	0%	10%	20%	30%	40%	50%	60%	70%	80%	90%	100%	N/A
%	◯	◯	◯	◯	◯	◯	◯	◯	◯	◯	◯	◯

4. Considérant son application, la connaissance du public, et les pratiques exemplaires, comment évalueriez-vous l'étape d'apprenti conducteur de votre province?
Les pratiques exemplaires en vigueur pour l'étape d'apprenti conducteur suggèrent:
• une période d'au moins 12 mois
• permission écrite d'un parent ou d'un tuteur légal
• surveillance parentale stricte à tout moment
• transition à l'étape intermédiaire seulement si aucune violation des conditions pendant cette étape
L'échelle d'évaluation globale est 0 = pas bon , 5 = moyen et 10 = extrêmement bon

	0	1	2	3	4	5	6	7	8	9	10	N/A
Le permis d'apprenti conducteur	◯	◯	◯	◯	◯	◯	◯	◯	◯	◯	◯	◯

Autres commentaires (facultatif)

3. L'étape intermédiaire

Programmes d'obtention du permis de conduire par étapes (PCÉ)

1. Votre province a-t-elle une étape intermédiaire dans son programme de permis de conduire par étapes?

- Oui
- Non
- Ne sais pas

2. Si oui, sur la base de votre expérience ou de votre expertise est-ce que cette étape intermédiaire est strictement imposée?

- Oui
- Non
- Ne sais pas
- Ne s'applique pas

3. Selon votre meilleure estimation, quel pourcentage du public connaît l'étape intermédiaire?

	0%	10%	20%	30%	40%	50%	60%	70%	80%	90%	100%	N/A
%												

4. Considérant son application, la connaissance du public, et les pratiques exemplaires, comment évalueriez-vous l'étape intermédiaire du PCÉ de votre province?

Les pratiques exemplaires en vigueur pour l'étape intermédiaire suggèrent:
• progression à l’étape de plein permis seulement si aucune violation pendant l’étape d’apprenti conducteur et pendant l’étape intermédiaire
• en cas d' accident avec faute du conducteur ou d'une accusation, le jeune conducteur doit prendre un cours de perfectionnement de conduite ou une mesure semblable d'amélioration de conduite avant de passer vers l’étape de plein permis
• au moins 50 heures de pratique (incluant la conduite de nuit) certifiées par un système de carnet avant de compléter cette étape

L'échelle d'évaluation globale est 0 = pas bon , 5 = moyen et 10 = extrêmement bon

	0	1	2	3	4	5	6	7	8	9	10	N/A
L’étape intermédiaire	○	○	○	○	○	○	○	○	○	○	○	○

Autres commentaires (facultatif)

4. L’alcoolémie

1. La loi du PCÉ de votre province inclut-elle des restrictions pour le taux d’alcool dans le sang permis?

○ Oui

○ Non

○ Ne sais pas

Programmes d'obtention du permis de conduire par étapes (PCÉ)

2. Si oui, sur la base de votre expérience ou de votre expertise est-ce que le taux d'alcool dans le sang permis est strictement imposé?

- Oui
- Non
- Ne sais pas
- Ne s'applique pas

3. Selon votre meilleure estimation, quel pourcentage du public connaît les restrictions du taux d'alcool dans le sang permis?

	0%	10%	20%	30%	40%	50%	60%	70%	80%	90%	100%	N/A
%	○	○	○	○	○	○	○	○	○	○	○	○

4. Considérant son application, la connaissance du public, et les pratiques exemplaires, comment évalueriez-vous vos règlements provinciaux de PCÉ au regard des restrictions du taux d'alcool dans le sang permis?

Les pratiques exemplaires suggèrent une alcoolémie égale à zéro (aucune trace d'alcool en conduisant).

L'échelle d'évaluation globale est 0 = pas bon , 5 = moyen et 10 = extrêmement bon

	0	1	2	3	4	5	6	7	8	9	10	N/A
L'alcoolemie	○	○	○	○	○	○	○	○	○	○	○	○

Autres commentaires (facultatif)

5. L'obligation de porter la ceinture de sécurité

1. La loi du PCÉ de votre province inclut-elle l'obligation du port de la ceinture de sécurité?

- Oui
- Non
- Ne sais pas

2. Si oui, sur la base de votre expérience ou de votre expertise est-ce que l'obligation du port de la ceinture de sécurité est strictement imposée chez les jeunes conducteurs?

- Oui
- Non
- Ne sais pas
- Ne s’applique pas

3. Selon votre meilleure estimation, quel pourcentage du public connaît cette obligation de porter la ceinture de sécurité?

	0%	10%	20%	30%	40%	50%	60%	70%	80%	90%	100%	N/A
%	○	○	○	○	○	○	○	○	○	○	○	○

4. Considérant leur application, la connaissance du public, et les pratiques exemplaires, comment évalueriez-vous vos règlements provinciaux de PCÉ quant à l'obligation de porter la ceinture de sécurité?

Les pratiques exemplaires suggèrent l'obligation de porter la ceinture de sécurité.

L'échelle d'évaluation globale est 0 = pas bon , 5 = moyen et 10 = extrêmement bon

	0	1	2	3	4	5	6	7	8	9	10
L’obligation de porter la ceinture de sécurité	○	○	○	○	○	○	○	○	○	○	○

Autres commentaires (facultatif)

6. Le couvre-feu pour la conduite de nuit

1. La loi du PCÉ de votre province inclut-elle un couvre-feu pour la conduite de nuit?

- Oui
- Non
- Ne sais pas

Programmes d'obtention du permis de conduire par étapes (PCÉ)

2. Si oui, sur la base de votre expérience ou de votre expertise est-ce que le couvre-feu pour la conduite de nuit est strictement imposé?

- Oui
- Non
- Ne sais pas
- Ne s'applique pas

3. Selon votre meilleure estimation, quel pourcentage du public connaît l'obligation du couvre-feu pour la conduite de nuit?

	0%	10%	20%	30%	40%	50%	60%	70%	80%	90%	100%	N/A
%	○	○	○	○	○	○	○	○	○	○	○	○

4. Considérant son application, la connaissance du public, et les pratiques exemplaires, comment évalueriez-vous vos règlements provinciaux de PCÉ quant au couvre-feu pour la conduite de nuit?

Les pratiques exemplaires suggèrent que la loi du PCÉ interdise la conduite de nuit non supervisée minimalement entre 22 h et 5 h.

L'échelle d'évaluation globale est 0 = pas bon , 5 = moyen et 10 = extrêmement bon

	0	1	2	3	4	5	6	7	8	9	10	N/A
Le couvre-feu pour la conduite de nuit	○	○	○	○	○	○	○	○	○	○	○	○

Autres commentaires (facultatif)

7. Les restrictions pour le nombre de passagers

1. La loi du PCÉ de votre province limite-t-elle le nombre de passagers pour les jeunes conducteurs?

- Oui
- Non
- Ne sais pas

2. Si oui, sur la base de votre expérience ou de votre expertise est-ce que les restrictions dans nombre de passager sont strictement imposées?

- Oui
- Non
- Ne sais pas
- Ne s’applique pas

3. Selon votre meilleure estimation, quel pourcentage du public connaît des restrictions du nombre de passagers?

	0%	10%	20%	30%	40%	50%	60%	70%	80%	90%	100%	N/A
%	○	○	○	○	○	○	○	○	○	○	○	○

4. Considérant leur application, la connaissance du public, et les pratiques exemplaires, comment évalueriez-vous vos règlements provinciaux de PCÉ quant aux restrictions du nombre passagers?

Les pratiques exemplaires suggèrent une limite dans le nombre de passagers adolescents qui peuvent accompagner le conducteur adolescent.

L'échelle d'évaluation globale est 0 = pas bon , 5 = moyen et 10 = extrêmement bon

	0	1	2	3	4	5	6	7	8	9	10	N/A
Les restrictions pour le nombre de passagers	○	○	○	○	○	○	○	○	○	○	○	○

Autres commentaires (facultatif)

8. Les restrictions quant à l’utilisation de téléphones cellulaires

1. La loi du PCÉ de votre province a-t-elle des restrictions quant à l’utilisation de téléphones cellulaires?

- Oui
- Non
- Ne sais pas

Programmes d’obtention du permis de conduire par étapes (PCÉ)

2. Si oui, sur la base de votre expérience ou de votre expertise est-ce que les restrictions quant à l’utilisation des téléphones cellulaires sont strictement imposées?

- ◯ Oui
- ◯ Non
- ◯ Ne sais pas
- ◯ Ne s’applique pas

3. Selon votre meilleure estimation, quel pourcentage du public connaît les restrictions quant à l’utilisation des téléphones cellulaires?

	0%	10%	20%	30%	40%	50%	60%	70%	80%	90%	100%	N/A
%	◯	◯	◯	◯	◯	◯	◯	◯	◯	◯	◯	◯

4. Considérant leur application, la connaissance du public, et les pratiques exemplaires, comment évalueriez-vous vos règlements provinciaux de PCÉ quant aux restrictions d’utilisation de téléphones cellulaires?

Les pratiques exemplaires suggèrent l'interdiction d’utilisation de téléphones cellulaires et de messagerie texte.

L'échelle d'évaluation globale est 0 = pas bon , 5 = moyen et 10 = extrêmement bon

	0	1	2	3	4	5	6	7	8	9	10	N/A
L'interdiction quant à l’utilisation de téléphones cellulaires et de messagerie texte	◯	◯	◯	◯	◯	◯	◯	◯	◯	◯	◯	◯

Autres commentaires (facultatif)

9. Signes/plaques “A” (pour apprenti) et “N” (pour nouveau conducteur)

Programmes d’obtention du permis de conduire par étapes (PCÉ)

1. La loi du PCÉ de votre province exige-t-elle l'utilisation de signes / plaques pour indiquer les « apprentis » et les « nouveaux conducteurs »?

- Oui
- Non
- Ne sais pas

2. Si oui, sur la base de votre expérience ou de votre expertise est-ce que l'utilisation des signes/plaques pour indiquer les « apprentis » et les « nouveaux conducteurs » est strictement imposée?

- Oui
- Non
- Ne sais pas
- Ne s’applique pas

3. Selon votre meilleure estimation, quel pourcentage du public connaît l’exigence des signes/plaques pour indiquer les « apprentis » et les « nouveaux conducteurs »?

	0%	10%	20%	30%	40%	50%	60%	70%	80%	90%	100%	N/A
%	○	○	○	○	○	○	○	○	○	○	○	○

4. Considérant leur application, la connaissance du public, et les pratiques exemplaires, comment évalueriez-vous vos règlements provinciaux de PCÉ quant à l'utilisation des signes/plaques pour indiquer les « apprentis » et les « nouveaux conducteurs »?

Il n’y a pas de pratiques exemplaires.

L'échelle d'évaluation globale est 0 = pas bon , 5 = moyen et 10 = extrêmement bon

	0	1	2	3	4	5	6	7	8	9	10	N/A
Les signes/plaques	○	○	○	○	○	○	○	○	○	○	○	○

Autres commentaires (facultatif)

10. La réduction de temps par des cours de conduite

Programmes d’obtention du permis de conduire par étapes (PCÉ)

1. Le règlement du PCÉ de votre province permet-elle la réduction de la période d’apprenti conducteur ou de l'étape intermédiaire pour les jeunes conducteurs qui complètent un cours de conduite?

- ◯ Oui
- ◯ Non
- ◯ Ne sais pas

2. Selon les pratiques exemplaires, comment évalueriez-vous vos règlements provinciaux de PCÉ quant à la réduction de la période d’apprenti suite à des cours de conduite?

Les pratiques exemplaires suggèrent de n'avoir AUCUNE réduction de temps pour les cours de conduite.

L'échelle d'évaluation globale est 0 = pas bon , 5 = moyen et 10 = extrêmement bon

	0	1	2	3	4	5	6	7	8	9	10	N/A
Réduction du temps	◯	◯	◯	◯	◯	◯	◯	◯	◯	◯	◯	◯

Autres commentaires (facultatif)

11. ÉVALUATION GLOBALE

1. Basé sur vos réponses précédentes, évaluez svp la loi du PCÉ de votre province globalement.

L'échelle d'évaluation globale est 0 = pas bon , 5 = moyen et 10 = extrêmement bon

	0	1	2	3	4	5	6	7	8	9	10
Évaluation globale	◯	◯	◯	◯	◯	◯	◯	◯	◯	◯	◯

Autres commentaires (facultatif)

12. Merci

Merci d’avoir contribuer à une meilleure compréhension des lois de PCÉ au Canada. Votre participation

est très appréciée!

Booster Seat Survey

The Canadian Injury Indicators Team, funded by the Canadian Institutes of Health Research (CIHR), is attempting to understand the best practice elements that are outlined in booster seat laws in different provinces in Canada. The objective of this survey is to evaluate the components of provincial booster seat legislation, including enforcement and public awareness, against evidence-based best practices. The results of this survey will be used to improve the effectiveness of provincial booster seat legislation in reducing child crash-related injuries and fatalities in Canada. In this survey, you will find a series of statements about booster seat best practices. Please rate your provincial/territorial legislation against each best practice statement.

Page #1

Instructions/Demographics

Q1 - As it relates to road safety, do you regard yourself as a practitioner, policy maker, researcher or physician?

- Practitioner
- Policy Maker
- Researcher
- Physician
- Other, please specify: ____________________________

Q2 - In which province/territory do you work?

- British Columbia
- Alberta
- Saskatchewan
- Manitoba
- Ontario
- Quebec
- New Brunswick
- Nova Scotia
- Prince Edward Island
- Newfoundland/Labrador
- Northwest Territories
- Yukon
- Nunavut

Q3 - Please select the choice that best corresponds to you.

- Have a special interest in the area of booster seat legislation
- Primarily work in the area of road safety
- Have a job related to the area of road safety and also have a special interest in booster seat legislation
- Other, please specify: ____________________________

Q4 - What proportion of your work day do you spend on booster seat legislation?

Note: 0 equals NO time spent on this issue, 5 equals half of work time spent on this issue, 10 equals all work time spent on this issue

- 0
- 1
- 2
- 3
- 4
- 5
- 6
- 7
- 8
- 9
- 10

Q5 - Which sector do you work in?

- Government
- University/College

- Hospital and/or Public Health
- Recreation and Sport
- Other, please specify: ____________________________

Q6 - What is the scope of your work?

- Provincial/Territorial
- Federal
- Both
- Other, please specify: ____________________________

Q7 - What is your job title?

____

Booster Seat Law

Q8 - Does your province have a booster seat law?

- Yes
- No
- Don't know

Q9 - Is there public awareness/education that this law exists?

- Yes
- No
- Don't know
- N/A

Q10 - If yes, in your best estimate, what percentage of the public is currently aware of the law?

- 0%
- 10%
- 20%
- 30%
- 40%
- 50%
- 60%
- 70%
- 80%
- 90%
- 100%
- N/A
- Further Comments (Optional): ___________________________

Age and Weight Stipulations

Best practice requires all children to be in an age, weight, and size appropriate restraint.

Q11 - Does your province's booster seat law have this component?

- Yes
- No
- Don't know

Q12 - If your answer is yes, then in your experience or expert opinion is this specific component strictly enforced?

- Yes
- No
- Don't know
- N/A

Q13 - Is there public awareness/education specific to this component?

- Yes
- No
- Don't know

Q14 - Considering the best practices and your responses on this component, please rate your province's booster seat law on a scale of 0-10.

The overall rating scale is 0 = not very good, 5 = average and 10 = extremely good.

- 0
- 1
- 2
- 3
- 4
- 5
- 6
- 7
- 8
- 9
- 10
- N/A
- Further Comments (Optional): ___________________________

Stipulations for Children in the Back Seat

Best practice requires children under 12 years old to be seated in the rear seat.

Q15 - Does your province’s booster seat law have this component?

- Yes
- No
- Don't know
- Other, please specify: ____________________________

Q16 - If your answer is yes, then in your experience or expert opinion is this specific component strictly enforced?

- Yes
- No
- Don't know
- N/A

Q17 - Is there public awareness/education specific to this component?

- Yes
- No
- Don't know

Q18 - Considering the best practices and your responses on this component, please rate your province’s booster seat law on a scale of 0-10.

The overall rating scale is 0 = not very good, 5 = average and 10 = extremely good.

- 0
- 1
- 2
- 3
- 4
- 5
- 6
- 7
- 8
- 9
- 10
- N/A
- Further Comments (Optional): ____________________________

No Legislation Exemptions

Best practice requires that no vehicle is exempt from booster seat legislation (e.g. Taxis and rental vehicles).

Q19 - Does your province's booster seat law have this component?

- Yes
- No
- Don't know
- Other, please specify: ___________________________

Q20 - If your answer is yes, then in your experience or expert opinion is this specific component strictly enforced?

- Yes
- No
- Don't know
- N/A

Q21 - Is there public awareness/education specific to this component?

- Yes
- No
- Don't know

Q22 - Considering the best practices and your responses on this component, please rate your province's booster seat law on a scale of 0-10.

The overall rating scale is 0 = not very good, 5 = average and 10 = extremely good.

- 0
- 1
- 2
- 3
- 4
- 5
- 6
- 7
- 8
- 9
- 10
- N/A
- Further Comments (Optional): ___________________________

Driver's Responsibility

Best practice states that the driver should be responsible for ensuring that children are placed in age and size appropriate restraints.

Q23 - Does your province’s booster seat law have this component?

- Yes
- No
- Don't know

Q24 - If your answer is yes, then in your experience or expert opinion is this specific component strictly enforced?

- Yes
- No
- Don't know
- N/A

Q25 - Is there public awareness/education specific to this component?

- Yes
- No
- Don't know

Q26 - Considering the best practices and your responses on this component, please rate your province’s booster seat law on a scale of 0-10.

The overall rating scale is 0 = not very good, 5 = average and 10 = extremely good.

- 0
- 1
- 2
- 3
- 4
- 5
- 6
- 7
- 8
- 9
- 10
- N/A
- Further Comments (Optional): ___________________________

Non-Compliance Actions

Best practice requires that both a fine and driver license demerit points are given for non-compliance to legislation.

Q27 - Does your province’s booster seat law have this component?

- Yes
- No
- Don't know
- Other, please specify: ___________________________

Q28 - If your answer is yes, then in your experience or expert opinion is this specific component strictly enforced?

- Yes
- No
- Don't know
- N/A

Q29 - Is there public awareness/education specific to this component?

- Yes
- No
- Don't know

Q30 - Considering the best practices and your responses on this component, please rate your province’s booster seat law on a scale of 0-10.

The overall rating scale is 0 = not very good, 5 = average and 10 = extremely good.

- 0
- 1
- 2
- 3
- 4
- 5
- 6
- 7
- 8
- 9
- 10
- N/A
- Further Comments (Optional): ___________________________

Incentive Programs

Best practice has shown that booster seat incentives or giveaway programs combined with education (e.g. financial incentives, free booster seats) are effective at increasing usage of booster seats.

Q31 - Does your province currently have booster seat incentives or giveaway program combined with education?

- Yes
- No
- Don't know
- Other, please specify: ___________________________

Q32 - Does the public know that these programs exist?

- Yes
- No
- Don't know
- N/A

Q33 - Considering the best practices and your responses on this component, please rate your province's booster seat law on a scale of 0-10.

The overall rating scale is 0 = not very good, 5 = average and 10 = extremely good.

- 0
- 1
- 2
- 3
- 4
- 5
- 6
- 7
- 8
- 9
- 10
- N/A
- Further Comments (Optional): ___________________________

Additional Questions

Q34 - Does your province have appropriate booster seat use guidance clinics/telephone hot lines for parents and care givers?

- Yes
- No
- Don't know
- Other, please specify: ____________________________

Q35 - Does your province have observational surveys in addition to Transport Canada's Child Restraint Use Survey?

- Yes
- No
- Don't know
- Other, please specify: ____________________________

Overall Rating

Q36 - Considering your responses to this survey, please rate your province's booster seat legislation overall.

The overall rating scale is 0 = not good, 5 = average and 10 = extremely good.

- 0
- 1
- 2
- 3
- 4
- 5
- 6
- 7
- 8
- 9
- 10
- N/A
- Further Comments (Optional): ___________________________

Thank you for contributing to an understanding of booster seat legislation in Canada. Your participation in the survey is greatly appreciated!

Sondage sur l'utilisation des sièges d'auto pour enfants

L'Équipe Canadienne sur les indicateurs des blessures (Canadian Injury Indicators Team), financée par les Instituts de Recherche en Santé du Canada (IRSC), tente de mieux comprendre quelles composantes des pratiques exemplaires sur l'utilisation des sièges d'auto pour enfants sont incluses dans les lois des différentes provinces du Canada. L'objectif de ce sondage est d'évaluer les composantes des législations provinciales à ce sujet, incluant leur application et la sensibilisation du public à l'endroit des pratiques exemplaires. Les résultats de ce sondage pourront contribuer à améliorer l'efficacité de la législation provinciale sur l'utilisation des sièges d'auto pour enfants, contribuant donc à réduire les blessures et les décès associés aux accidents d'automobiles au Canada. Dans ce sondage, vous trouviez une série d'énoncés en lien avec les pratiques exemplaires d'utilisation des sièges d'auto pour enfants. Nous vous prions donc d'évaluer votre législation provinciale/territoriale à la lumière de chaque énoncé.

Page #1

Introduction/données démographiques

Q1 - En ce qui concerne la sécurité routière, vous considérez-vous comme un praticien, un décideur politique, un chercheur ou un médecin?

- ☐ Praticien
- ☐ Décideur politique
- ☐ Chercheur
- ☐ Médecin
- ☐ Autre (spécifiez svp): ___________________________

Q2 - Dans quelle province/territoire travaillez-vous?

- ☐ Colombie-Britannique
- ☐ Alberta
- ☐ Saskatchewan
- ☐ Manitoba
- ☐ Ontario
- ☐ Québec
- ☐ Nouveau-Brunswick
- ☐ Nouvelle-Écosse
- ☐ Île-du-Prince-Édouard
- ☐ Terre-Neuve et Labrador
- ☐ Les Territoires du Nord-Ouest
- ☐ Yukon
- ☐ Nunavut

Q3 - Sélectionnez s'il vous plaît la case qui vous décrit le mieux:

- ☐ Vous avez un intérêt particulier pour le secteur de la législation des sièges d'auto pour enfantsr seat legislation
- ☐ Vous travaillez principalement dans le secteur de la sécurité routière
- ☐ Vous avez un emploi dans le secteur de la sécurité routière et avez également un intérêt particulier pour la législation des sièges d'auto pour enfants and also have a special interest in booster seat legislation
- ☐ Autre (spécifiez svp): ___________________________

Q4 - Quelle proportion de votre journée de travail consacrez-vous à la législation des sièges d'auto pour enfants?

Note : 0 signifie AUCUNE, 5 signifie la moitié du temps de travail, 10 signifie que toutes vos heures de travail sont consacrées a ce sujet.

- ○ 0
- ○ 1
- ○ 2
- ○ 3
- ○ 4
- ○ 5
- ○ 6
- ○ 7
- ○ 8
- ○ 9
- ○ 10

Q5 - Dans quel secteur travaillez-vous?

- Gouvernement
- Universitaire
- Hôpital et/ou santé publique
- Loisir et sport
- Autre (spécifiez svp): ___________________________

Q6 - Quelle est la portée de votre travail?

- Provinciale/territoriale
- Fédérale
- Les deux
- Autre (spécifiez svp): ___________________________

Q7 - Quel est votre titre d’emploi?

____

La loi sur l'utilisation des sièges d'auto pour enfants

Q8 - Votre province a-t-elle une loi sur l'utilisation des sièges d'auto pour enfants?

- Oui
- Non
- Ne sais pas

Q9 - Y a-t-il des mesures de sensibilisation du public/d'éducation à propos de l'existence de cette loi?

- Oui
- Non
- Ne sais pas
- Ne s'applique pas

Q10 - Si oui, quelle proportion de la population estimez-vous être au courant de la loi ?

- 0%
- 10%
- 20%
- 30%
- 40%
- 50%
- 60%
- 70%
- 80%
- 90%
- 100%
- N/A
- Autres commentaires (facultatif): ___________________________

L'exigences de poids et d'age

Les pratiques exemplaires exigent que tous enfants soient dans un dispositif de sécurité approprié pour leur âge, leur poids et leur taille.

Q11 - Est-ce que la loi sur l'utilisation des sièges d'auto pour enfants de votre province inclut cette composante?

- Oui
- Non
- Ne sais pas

Q12 - Si oui, d'après votre expérience où votre expertise, cette composante est-elle appliquée?

- Oui
- Non
- Ne sais pas
- Ne s'applique pas

Q13 - Y a-t-il des mesures de sensibilisation du public/d'éducation à propos de cette composante?

- Oui
- Non
- Ne savez pas

Q14 - En songeant aux pratiques exemplaires et à vos réponses au sujet de cette composante, s'il vous plait évaluez la loi sur l'utilisation des sièges d'auto pour enfants de votre province sur une échelle de 0-10.

Note : Dans l'échelle d'évaluation globale 0 = pas très bon, 5 = moyen, et 10 = extrêmement bon.

- 0
- 1
- 2
- 3
- 4
- 5
- 6
- 7
- 8
- 9
- 10
- N/A
- Autres commentaires (facultatif): ___________________________

L'exigences pour les Enfants sur le Siege Derrière

Les pratiques exemplaires exigent que tous enfants de moins de 12 ans s’asseoient sur la banquette arrière d’une auto.

Q15 - Est-ce que la loi sur l’utilisation des sièges d’auto pour enfants de votre province inclut cette composante?

- Oui
- Non
- Ne sais pas
- Autre (spécifiez svp): ___________________________

Q16 - Si oui, d’après votre expérience où votre expertise, cette composante est-elle appliquée?

- Oui
- Non
- Ne sais pas
- Ne s’applique pas

Q17 - Y a-t-il des mesures de sensibilisation du public/d’éducation à propos de l’existence de cette composante?

- Oui
- Non
- Ne savez pas

Q18 - En songeant aux pratiques exemplaires et à vos réponses au sujet de cette composante, s’il vous plait évaluez la loi sur l’utilisation des sièges d’auto pour enfants de votre province sur une échelle de 0-10.

Note : Dans l'échelle d'évaluation globale 0 = pas très bon, 5 = moyen, et 10 = extrêmement bon.

- 0
- 1
- 2
- 3
- 4
- 5
- 6
- 7
- 8
- 9
- 10
- N/A
- D'autres commentaires (facultatif): ___________________________

Pas d’exonérations de législation

Les pratiques exemplaires exigent qu’aucun véhicule ne soit exempté de la législation des sièges d’auto pour enfants (p. ex. taxis et voitures de location)

Q19 - Est-ce que la loi sur l’utilisation des sièges d’auto pour enfants de votre province inclut cette composante?

- Oui
- Non
- Ne sais pas
- Autre (spécifiez svp): ___________________________

Q20 - Si oui, d’après votre expérience où votre expertise, cette composante est-elle appliquée?

- Oui
- Non
- Ne sais pas
- Ne s’applique pas

Q21 - Y a-t-il des mesures de sensibilisation du public/d’éducation à propos de l’existence de cette composante?

- Oui
- Non
- Ne savez pas

Q22 - En songeant aux pratiques exemplaires et à vos réponses au sujet de cette composante, s’il vous plait évaluez la loi sur l’utilisation des sièges d’auto pour enfants de votre province sur une échelle de 0-10.

Note : Dans l'échelle d'évaluation globale 0 = pas très bon, 5 = moyen, et 10 = extrêmement bon.

- 0
- 1
- 2
- 3
- 4
- 5
- 6
- 7
- 8
- 9
- 10
- N/A
- D'autres commentaires (facultatif): ___________________________

La responsabilité du conducteur

Les pratiques exemplaires exigent que le conducteur ait la responsabilité de placer tous enfants dans des dispositifs de sécurité appropriés à leur âge et leur taille.

Q23 - Est-ce que la loi sur l'utilisation des sièges d'auto pour enfants de votre province inclut cette composante?

- Oui
- Non
- Ne sais pas

Q24 - Si oui, d'après votre expérience où votre expertise, cette composante est-elle appliquée?

- Oui
- Non
- Ne sais pas
- Ne s'applique pas

Q25 - Y a-t-il des mesures de sensibilisation du public/d'éducation à propos de l'existence de cette composante?

- Oui
- Non
- Ne savez pas

Q26 - En songeant aux pratiques exemplaires et à vos réponses au sujet de cette composante, s'il vous plait évaluez la loi sur l'utilisation des sièges d'auto pour enfants de votre province sur une échelle de 0-10.

Note: Dans l'échelle d'évaluation globale 0 = pas très bon, 5 = moyen, et 10 = extrêmement bon.

- 0
- 1
- 2
- 3
- 4
- 5
- 6
- 7
- 8
- 9
- 10
- N/A
- D'autres commentaires (facultatif): ___________________________

Actions de non-respect de la loi

Les pratiques exemplaires exigent qu'une amende ainsi que des points d'inaptitude soient imposés pour le non-respect de la loi.

Q27 - Est-ce que la loi sur l'utilisation des sièges d'auto pour enfants de votre province inclut cette composante?

- Oui
- Non
- Ne sais pas
- Autre (spécifiez svp): ___________________________

Q28 - Si oui, d'après votre expérience où votre expertise, cette composante est-elle appliquée?

- Oui
- Non
- Ne sais pas
- Ne s'applique pas

Q29 - Y a-t-il des mesures de sensibilisation du public/d'éducation à propos de l'existence de cette composante?

- Oui
- Non
- Ne savez pas

Q30 - En songeant aux pratiques exemplaires et à vos réponses au sujet de cette composante, s'il vous plait évaluez la loi sur l'utilisation des sièges d'auto pour enfants de votre province sur une échelle de 0-10.

Note: Dans l'échelle d'évaluation globale 0 = pas très bon, 5 = moyen, et 10 = extrêmement bon.

- 0
- 1
- 2
- 3
- 4
- 5
- 6
- 7
- 8
- 9
- 10
- N/A
- D'autres commentaires (facultatif): ___________________________

Programmes de motivation

Les pratiques exemplaires ont démontré que des incitatifs ou des programmes de distribution, conjugués à de l’éducation (ex. incitatifs financiers, sièges gratuits) sont efficaces pour augmenter l’usage des sièges d’auto pour enfants.

Q31 - Est-ce que votre province offre présentent des incitatifs en lien avec les sièges d’auto pour enfants ou des programmes de distribution, conjugués à de l’éducation?

- Oui
- Non
- Ne savez pas
- Autre (spécifiez svp): ____________________________

Q32 - Est-ce que le public sait que que ces programmes existent?

- Oui
- Non
- Ne savez pas
- Ne s’applique pas

Q33 - En songeant aux pratiques exemplaires et à vos réponses au sujet de cette composante, s’il vous plait évaluez la loi sur l’utilisation des sièges d’auto pour enfants de votre province sur une échelle de 0-10.

Note: Dans l'échelle d'évaluation globale 0 = pas très bon, 5 = moyen, et 10 = extrêmement bon.

- 0
- 1
- 2
- 3
- 4
- 5
- 6
- 7
- 8
- 9
- 10
- N/A
- D'autres commentaires (facultatif): ____________________________

Questions supplémentaires

Q34 - Votre province offre-t-elle des conseils sur l’utilisation appropriée des sièges d’auto pour enfants, sous forme de cliniques ou de lignes téléphoniques pour les parents et les aidants?

- Oui
- Non
- Ne savez pas
- Autre (spécifiez svp): ____________________________

Q35 - Votre province a-t-elle des sondages d’observation, autre que le sondage d’utilisation des dispositifs de retenue d'enfant de Transports Canada?

- Oui
- Non
- Ne savez pas
- Autre (spécifiez svp): ____________________________

Évaluation globale

Q36 - En songeant à vos réponses à ce sondage, s'il vous plait évaluez la loi sur l'utilisation des sièges d'auto pour enfants de votre province sur une échelle de 0-10.

Note: Dans l'échelle d'évaluation globale 0 = pas très bon, 5 = moyen, et 10 = extrêmement bon.

- 0
- 1
- 2
- 3
- 4
- 5
- 6
- 7
- 8
- 9
- 10
- N/A
- Further Comments (Optional): ____________________________

Nous vous remercions d’avoir contribué à une meilleure compréhension des lois sur l’utilisation des sièges d’auto pour enfants au Canada. Votre participation est très appréciée!
